# Supplementary material for: Takotsubo Syndrome during COVID-19 Pandemic in the Veneto Region, Italy
Source: Viruses. 2022 Sep 6;14(9):1971. doi: 10.3390/v14091971 (PMC9506046; doi:10.3390/v14091971)
Supplement: Supplementary file 1 [file viruses-14-01971-s001.zip › viruses-1863136-Supplementary.pdf]

Supplementary Table S1. Characteristics of the recruiting Hospitals.

Hospital	Type	Beds in Cardiology ward	Beds in ICCU	Beds converted for COVID-19 patients
Arzignano	Spoke	14	4	0
Mirano	Hub	32	8	0
Legnago	Hub	29	8	0
Padova	Hub	44	16	0
Rovigo	Hub	24	9	1
Mestre	Hub	29	8	0
San Bonifacio	Spoke	12	4	0
San Donà di Piave	Hub	22	8	0
Negrar	Hub	17	4	0
Bassano del Grappa	Hub	20	14	0
Venezia	Hub	18	7	0
Verona	Hub	44	8	0
Chioggia	Spoke	6	4	0
